# Intrapericardial Mature Cystic Teratoma

**DOI:** 10.1016/j.atssr.2026.02.018

**Published:** 2026-03-03

**Authors:** Madison A. Grasty, John S. Riley, Anna M. Moran, Leslie A. Litzky, Michael Ibrahim, Taine T.V. Pechet

**Affiliations:** 1 Department of General Surgery, Hospital of the University of Pennsylvania, Philadelphia, Pennsylvania; 2 Department of Pathology, Presbyterian Medical Center, Philadelphia, Pennsylvania; 3 Department of Cardiac Surgery, Presbyterian Medical Center, Philadelphia, Pennsylvania; 4 Department of Thoracic Surgery, Presbyterian Medical Center, Philadelphia, Pennsylvania

## Abstract

Mediastinal masses are an uncommon and encompass a wide variety of lesions. Their diagnosis and management are often dependent upon the radiographic features, symptoms, and serologies. Germ cell tumors are exceedingly rare in this location. This case report describes a mediastinal mass in a 37-year-old man who presented with occasional chest pain. Pathology revealed an intrapericardial mature cystic teratoma that arose from the adventitia of the aorta. Mature teratomas are most commonly found outside the pericardium. This report describes the exceedingly rare diagnosis of a mature cystic teratoma arising from the aortic adventitia.

Mediastinal masses describe a broad spectrum of pathologic entities that can occur in different mediastinal compartments. The most common pathologic lesions in the anterior mediastinal compartment are thymomas. However, lymphomas and germ cell tumors can also occur within this space with some regularity, as can benign lesions that are often cystic. Germ cell tumors are the result of aberrant growth of the germ layers (endoderm, mesoderm, and ectoderm) and are most commonly found in the gonads. When they are extragonadal, the most common site is the mediastinum. In the mediastinum they are often identifiable on initial imaging because of unique radiographic features. Teratomas are benign germ cell tumors typically located in the extrapericardial mediastinum. Intrapericardial teratomas are typically symptomatic and found during infancy, and resection is recommended. Fewer than 1% of mature teratomas in the adult are found intrapericardially.^1^ This report describes a case of a symptomatic intrapericardial mature cystic teratoma arising from the adventitia of the aorta in an adult.

This is a 37-year-old man who presented with intermittent chest pain. His past medical history was otherwise notable for depression, hemorrhoids, and smoking (cigarettes and marijuana). A chest radiograph was notable for widening of the superior mediastinum, and a chest computed tomography revealed a 4.5-cm complex mediastinal mass located anterior to the right main pulmonary artery and the superior vena cava (SVC) with significant SVC compromise (Figure 1). A treadmill stress test and transthoracic echocardiography were unremarkable, and pulmonary function testing was normal with a mildly reduced diffusion capacity for carbon monoxide of 77. The patient was referred to this institution, where cardiac magnetic resonance imaging (MRI) revealed a 2.7 x 3.5cm lobular, well-defined mass that was isodense on T1, hyperintense on T2, and demonstrated no signal change with fat saturation, first-pass perfusion, or late gadolinium enhancement. MRI characteristics favored a complex pericardial cyst causing significant SVC compression. The patient was experiencing more episodic dizziness, shortness of breath, and ongoing chest pain. There was no evidence of SVC syndrome on exam. Biopsy was deferred as imaging suggested the patient had a pericardial cyst or potentially a cystic thymoma. Surgical resection was planned with initial thoracoscopic exploration as it was thought the mass may be amenable to minimally invasive management.

**Figure 1:**
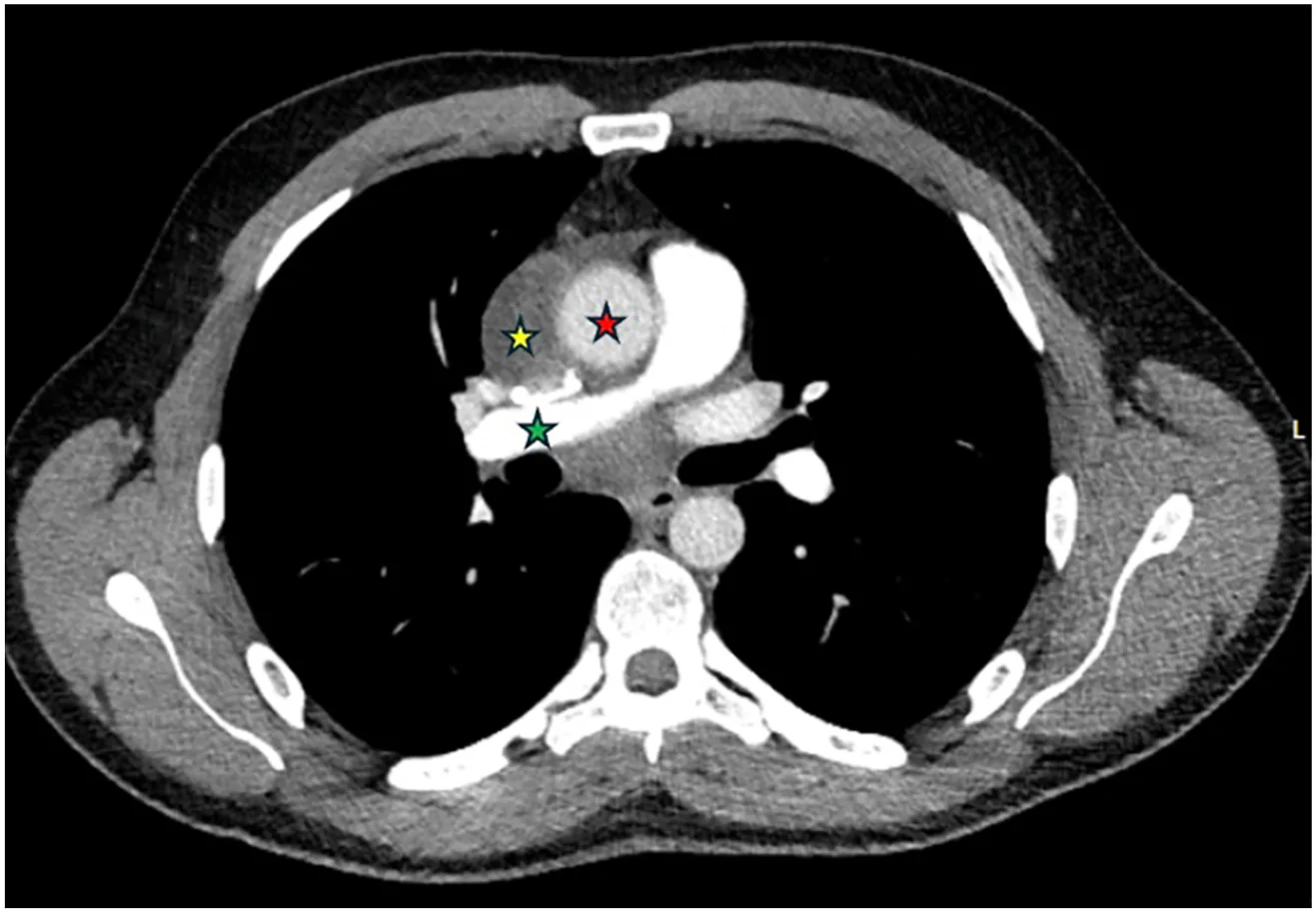
Chest computed tomography with a yellow star identifying the lesion abutting the lateral aspect of the ascending aorta (red star) and anterior to the right pulmonary artery (green star).

Thoracoscopy was performed through the fifth intercostal space in the anterior axillary line with subsequent ports placed in the second intercostal space and parasternally at the diaphragmatic reflection. It demonstrated that the mass was intrapericardial and intimately associated with the ascending aorta but free of the SVC and atrial appendage (Figure 2). A pericardiotomy was made and, upon manipulation, the mass was adherent to the adventitia of the ascending aorta. A sternotomy was performed. The lesion was carefully dissected free from the aorta using the electrocautery. It was completely resected without rupture or injury to the media of the aortic wall, thus leaving the media intact.

**Figure 2:**
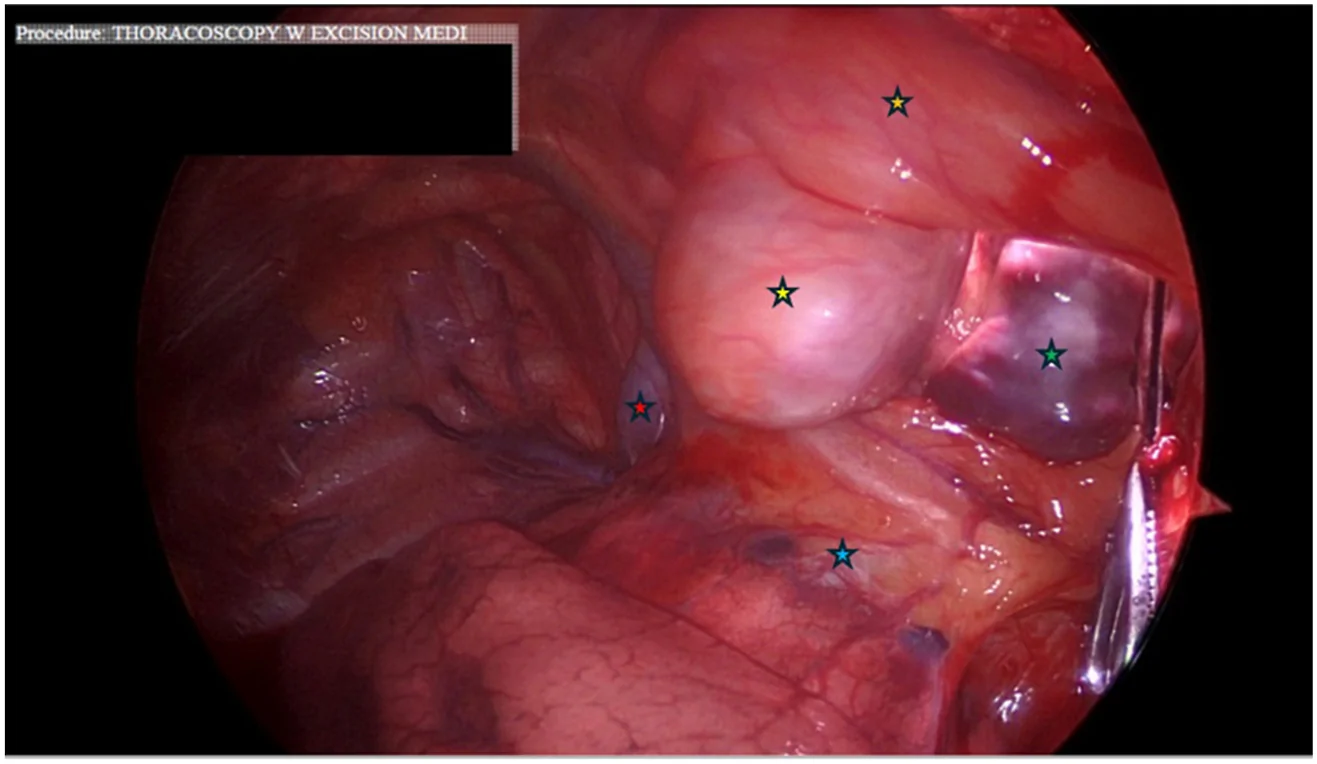
Intraoperative photo during thoracoscopy. The yellow star identifies the lesion, which was superior to the right atrial appendage (green star), anterior to the hilum of the right lung (blue star), inferior to the azygous vein (red star), and posterior to the pericardial fat pad (orange star).

Gross examination revealed an intact, well-circumscribed mass (Figure 3A), which when opened had cystic and solid components (Figure 3A). Frozen section demonstrated a cyst lined with respiratory epithelium favoring a thyroglossal duct cyst.

**Figure 3:**
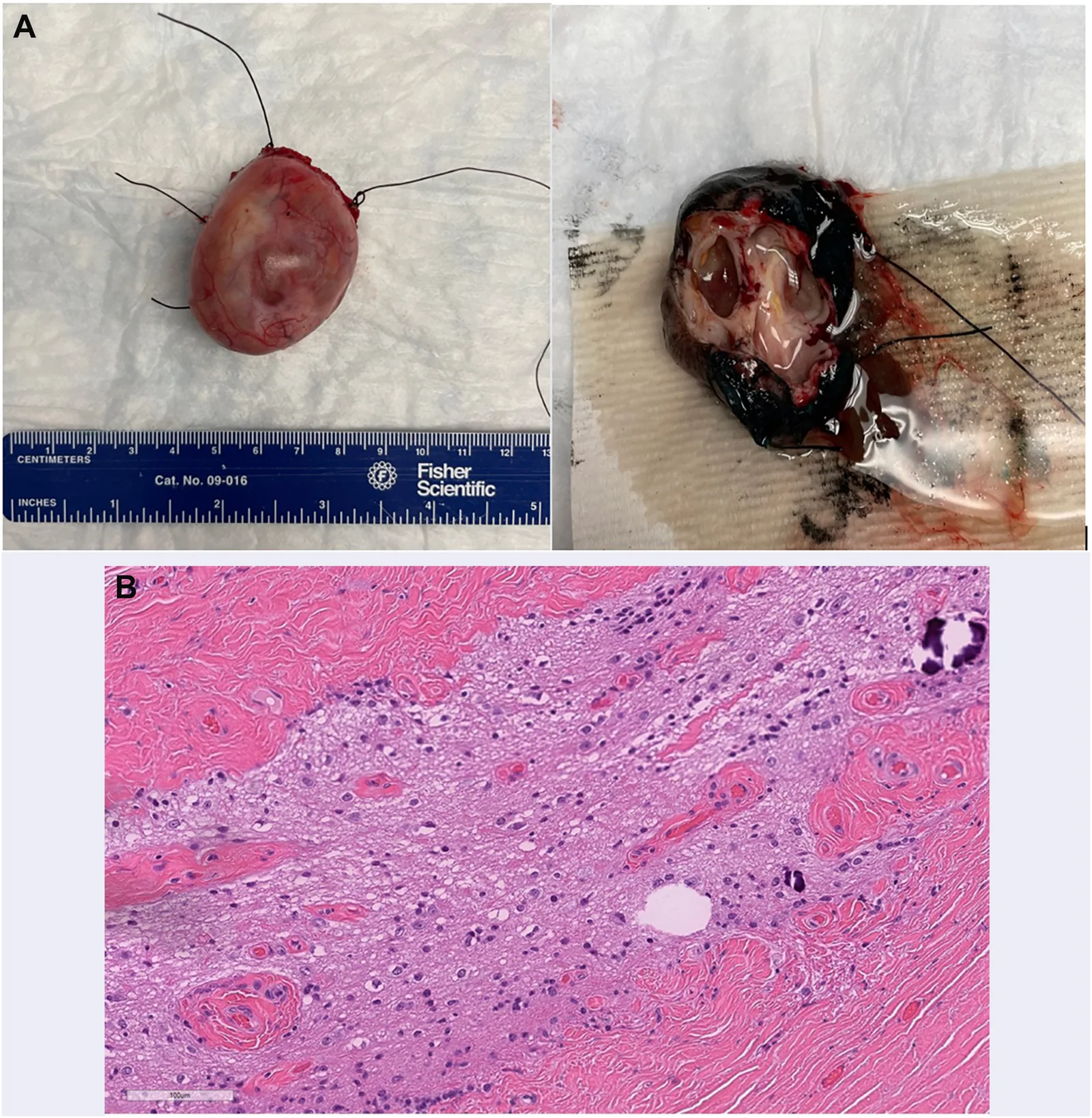
(A) Gross pathology of specimen, with the bisected specimen incised on the right. (B) Histology with findings of mature glial tissue.

The postoperative course was uneventful and the patient was discharged on postoperative day 2. Final pathology revealed a mature cystic teratoma with uninvolved margins (Figure 3B). A follow-up MRI 4 months postoperatively revealed no evidence of the lesion and no appreciable aortic wall abnormality. The patient’s preoperative symptoms had fully resolved.

## Comment

Germ cell tumors are known to arise from embryologic origins and can appear throughout the body. Mature cystic teratomas of the mediastinum, a benign form of germ cell tumor, are most common in young adults and typically are asymptomatic.^2^ This is also true of the intrapericardial subtype, which is historically asymptomatic except in the pediatric population.^1^^,^^3^^,^^4^ Reports describing fetal intrapericardial teratomas in infants with symptoms tend to highlight the associated hemodynamic compromise and hydrops fetalis, which are believed to arise due to mass effect impairing venous return and cardiac output.^4^ The most common location in one small case series was at the junction of the ascending aorta and right atrial appendage (the location of this patient’s lesion).^5^ In adults, intrapericardial mature cystic teratomas involving the aortic adventitia are extremely rare, with only 6 surgical cases identified in the literature.^1^^,^^3^^,^^6^ The symptomatic nature of this adult patient’s intrapericardial teratoma is even more unusual. Clinical follow-up confirmed resolution of the symptoms and the radiographic abnormality at 4 months.

Typical imaging features of a mature cystic teratoma on computed tomography are a well-defined mass with solid and cystic components that sometimes contain fat and calcium. On MRI, they most commonly have a heterogenous signal intensity, reflective of the variability of the components.^3^ Mature cystic teratomas have a small risk of malignant transformation to squamous cell carcinoma, with an incidence reported to be 0.17%-2%.^7^ The utility of tissue biopsy of intrapericardial teratomas is unknown.^1^ Mature cystic teratomas are not responsive to radiation or chemotherapy, and there is no role for neoadjuvant or adjuvant therapy.^8^

While computed tomography and MRI are key elements of the diagnostic evaluation, tissue sampling is often required to firmly establish the diagnosis. Given the location of many of these mediastinal lesions, and particularly intrapericardial tumors, an operative approach is typically necessary for pathologic diagnosis. The recommended treatment is surgical resection for diagnosis and therapy, due to the risk of malignant potential, compressive symptoms, rupture, and pericarditis.^1^^,^^3^ However the precise risk of malignant transformation is unknown given the scarcity of cases.

Mature cystic teratomas are a rare pathology, most commonly diagnosed in fetuses and children. This case is unique in reporting an adult with a symptomatic mature cystic teratoma in an intrapericardial location. Safe resection through a sternotomy was completed, and pathology did not reveal any malignant transformation. This case highlights the heterogeneity of mediastinal masses, their unique presentations, and the recommended management.
